# Exome Sequencing Identifies Compound Heterozygous Mutations in* SCN5A* Associated with Congenital Complete Heart Block in the Thai Population

**DOI:** 10.1155/2016/3684965

**Published:** 2016-11-28

**Authors:** Chuphong Thongnak, Pornprot Limprasert, Duangkamol Tangviriyapaiboon, Suchaya Silvilairat, Apichaya Puangpetch, Ekawat Pasomsub, Chonlaphat Sukasem, Wasun Chantratita

**Affiliations:** ^1^Division of Pharmacogenomics and Personalized Medicine, Department of Pathology, Faculty of Medicine Ramathibodi Hospital, Mahidol University, Bangkok, Thailand; ^2^Laboratory for Pharmacogenomics, Somdech Phra Debaratana Medical Center (SDMC), Ramathibodi Hospital, Bangkok, Thailand; ^3^Excelence Center for Genomic Medicine, Faculty of Medicine Ramathibodi Hospital, Mahidol University, Bangkok, Thailand; ^4^Human Genetics Unit, Department of Pathology, Faculty of Medicine, Prince of Songkla Univerisity, Hat Yai, Songkhla, Thailand; ^5^Rajanagarindra Institute of Child Development, Chiang Mai, Thailand; ^6^Division of Pediatric Cardiology, Department of Pediatrics, Faculty of Medicine, Chiang Mai University, Chiang Mai, Thailand; ^7^Virology Laboratory, Department of Pathology, Faculty of Medicine Ramathibodi Hospital, Mahidol University, Bangkok, Thailand

## Abstract

*Background*. Congenital heart block is characterized by blockage of electrical impulses from the atrioventricular node (AV node) to the ventricles. This blockage can be caused by ion channel impairment that is the result of genetic variation. This study aimed to investigate the possible causative variants in a Thai family with complete heart block by using whole exome sequencing.* Methods*. Genomic DNA was collected from a family consisting of five family members in three generations in which one of three children in generation III had complete heart block. Whole exome sequencing was performed on one complete heart block affected child and one unaffected sibling. Bioinformatics was used to identify annotated and filtered variants. Candidate variants were validated and the segregation analysis of other family members was performed.* Results*. This study identified compound heterozygous variants, c.101G>A and c.3832G>A, in the* SCN5A* gene and c.28730C>T in the* TTN* gene.* Conclusions*. Compound heterozygous variants in the* SCN5A* gene were found in the complete heart block affected child but these two variants were found only in the this affected sibling and were not found in other unaffected family members. Hence, these variants in the* SCN5A* gene were the most possible disease-causing variants in this family.

## 1. Introduction

Congenital heart block is an uncommon disorder that occurs in about 1 in 20,000 live births [1]. It is characterized by anatomical or functional impairment in the conduction system which is caused by blockage of electrical impulses from atrioventricular node (AV node) to the ventricles [2]. The severity of heart block ranges from first-degree in which electrical impulse to the AV node is slower than normal to third-degree or complete heart block in which electrical impulses from the atrium do not reach ventricles at all [3].

The conduction defect can be caused by a defective link between cardiomyocytes or by ion channel impairment that changes action potential shapes [4]. Inherited defects in cardiac conduction have been linked to genetic variants in several genes such as* SCN5A*,* SCN1B*,* KCNJ2*,* HCN4, NKX2-5, TBX5, LMNA,* and* ANKB* [4–9]. Among these genes,* SCN5A* has been frequently reported with various phenotypes [10].* SCN5A* encodes *α* subunit of the cardiac sodium channel (NaV1.5) which controls the flow of sodium ions into cells that is essential in generation and transmission of electrical impulses [11]. A nonfunctional protein, which is caused by a mutation in the* SCN5A* gene, reduces entrance of sodium into the cells that results in difficulty producing and transmitting electrical signals resulting in heart block [12].

Mutations in* SCN5A*, which lead to loss or gain of sodium channel function, are associated with a spectrum of cardiac diseases including Brugada syndrome, Long QT syndrome type 3, sick sinus syndrome, and progressive familial heart block [12–16].

With the advantages of next-generation sequencing especially whole exome sequencing that can explore the sequence of all exons in a single experiment, sequencing has been used in several studies for comprehensive and unbiased identification of causative variants of diseases in the last decade [17, 18]. Likewise, whole exome sequencing has been used in other cardiovascular related studies to identify disease-causing mutations of familial atrial septal defects [19].

This study aims to investigate the possible causative variants in a Thai family with complete heart block by using whole exome sequencing. A combined method of familial data, exome sequencing, bioinformatics, and segregation analysis was able to identify 6 variants in 5 genes in which 2 variants in* SCN5A* were the most plausible disease-causing variants for heart block.

## 2. Materials and Methods

### 2.1. Subjects

**Figure 1:**
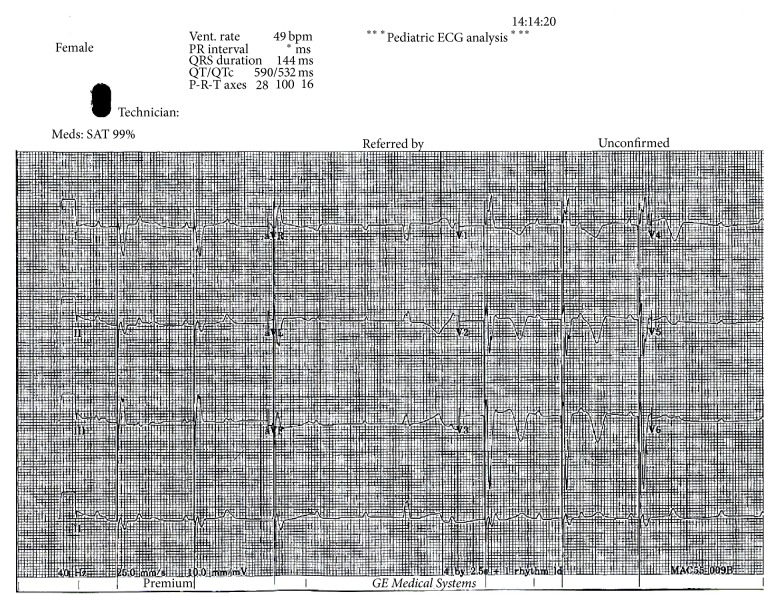
Electrocardiography of index case (III-2) shows third-degree atrioventricular block, atrial rate 100/min, ventricular rate 49/min, and ventricular pacing spikes before some QRS complexes.

The family in this study had 3 generations from which blood samples for DNA preparations were collected: grandmother (I-2), mother (II-2), and two children (III-2 and III-3). The II-2 was a single mother; therefore, we could not obtain blood for DNA from the father. The index case (III-2) had a third-degree AV block and had undergone cardiac surgery for epicardial pacemaker implantation at the age of 18 months. She was diagnosed with autism according to the DSM-IV at the age of 6 years. The other two siblings had no heart defects but one had learning disability (III-1, no DNA) and the other was autistic (III-3) (Figure 1). Extracted DNA samples from III-2 and III-3 were controlled for their quality by measuring DNA concentration using Nanodrop ND1000 and measuring fragmentation of DNA by agarose gel electrophoresis. These 2 samples were prepared for whole exome sequencing. DNA samples from other family members (I-2, II-2) were prepared for segregation analysis. Written informed consent was obtained from adult family members themselves and for all children and all procedures were approved by the Institutional Review Board (MURA2012/02/SN1) of the Faculty of Medicine Ramathibodi Hospital, Mahidol University, and EC48/364-006-3 of the Faculty of Medicine, Prince of Songkla University.

### 2.2. Exome Sequencing and Data Analysis

Whole exome sequencing was performed on family members III-2 and III-3. Samples were prepared following standard SOLID 5500xl (Applied Biosystems, California, USA) protocols for whole exome sequencing. Three micrograms of genomic DNA of each sample were fragmented using Covaris S2 (Covaris, Massachusetts, USA) and were captured for exome sequencing using TargetSeq Exome and Custom Enrichment System (Invitrogen, California, USA). The captured DNA was sequenced with 150 bp paired-end read on the SOLID 5500xl system according to the manufacturer's protocol. Primary analysis was performed on the sequencing machine using SOLID ICS software; then, raw sequenced data was transferred to the LifeScope Genomic Analysis server for secondary analysis. Sequence reads were mapped to the human reference genome assembly hg19 (GRCh37); then, variants calling for SNV, insertions, and deletions were performed.

Tertiary analysis was performed by using Golden Helix SVS software on identified candidate variants. Variants were filtered for minimum genotype quality of 20 and minimum coverage depths of 10 and then the qualified variants were annotated with the UCSC KnownGenes database to remove noncoding and synonymous variants. High frequency variants (minor allele frequencies greater than 2%) were excluded by annotation with allele frequencies from the 1000 Genomes Project Phase 3 [20] and an in-house exome database which consists of 172 Thai individuals. Deleterious protein functions were predicted using dbNSFP that compiled prediction scores from eleven prediction algorithms (SIFT, Polyphen2, LRT, MutationTaster, MutationAssessor, FATHMM, VEST3, CADD, MetaLR, MetaSVM, and PROVEAN) and 4 conservation scores, PhyloP, phastCons, GERP++, and SiPhy, and other related information [21]. Variants that were predicted not to alter protein function by any algorithm were excluded in this step. To narrow down variants, variants were focused on where these were located in the candidate genes list (Table 1). This genes list was created from combined known and suspected genes involved in the cardiovascular system from several sources and the cardiovascular defective candidate genes from the Enlis Genome Research software (Enlis, Berkeley, CA) gene panel. This genes list consists of 359 genes in total. Lastly, variants were filtered by their genotype. A genotype that was only in family member III-2 was indicated as a candidate variant for heart block. The summarized variants filter steps are shown in Figure 3.

Additionally, pathogenic variants which were related to other diseases followed were explored in The American College of Medical Genetics and Genomics (ACMG) recommendations for reporting of incidental findings in clinical exome and genome sequencing [22]. Known pathogenic variants were identified by using The Human Gene Mutation Database (HGMD) [23].

### 2.3. Variant Validations and Segregation Analysis

Sanger sequencing was used to validate the candidate variants found in whole exome sequencing and segregation analyses were performed on the family members. Primers were designed using the Primer3 version 0.4.0 web-based tool [24]. Sequencing reactions were performed using Applied Biosystems 3130 DNA Analyzer (Life Technologies, Carlsbad, CA, USA).

## 3. Results

A family with complete heart block in only one of the 3rd generation family members was explored. By whole exome sequencing, a total of 99,834 variants in family members III-2 and III-3 were detected with an average depth over 60x coverage. After removal of low quality, noncoding and synonymous variants, 14,284 variants remained. Subsequently, variants were reduced by a filtering pipeline that included variants with minor allele frequencies, inheritance models, and a candidate gene list which reduced the number of variants to 36 variants in 28 genes. Finally, variants were prioritized and selected as candidate variants by annotated information from the deleterious protein function prediction database that resulted in 21 heart defective candidate variants in 18 genes. A list of candidate variants is shown in Table 2. All 21 candidate variants were then investigated for validation and segregation analysis. Six variants successfully passed this step while the other variants were dropped for 2 reasons: (1) a discordance between whole exome sequencing and Sanger sequencing or (2) variants found in the affected child being found in other unaffected family members except for two or more variants that were found in the same gene which indicated compound heterozygous inheritance. These 6 variants in 5 genes were identified as candidate variants for heart defects in this family. The list of final candidate variants is shown in Table 3.

**Figure 2:**
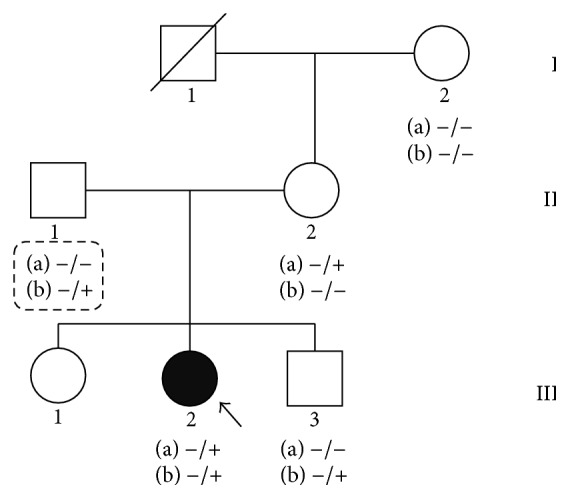
The lineage of the family with complete heart block indicated by the dark symbol, females by circles, and males by squares. Letters (a) and (b) indicate genotypes of 2 variants in the* SCN5A* gene for c.101G>A and c.3832G>A. The homozygous reference genotypes are indicated by (−/−) and heterozygous alternate genotypes by (−/+). The genotypes of II-1 are presumed genotypes which are inferred from his children.

**Figure 3:**
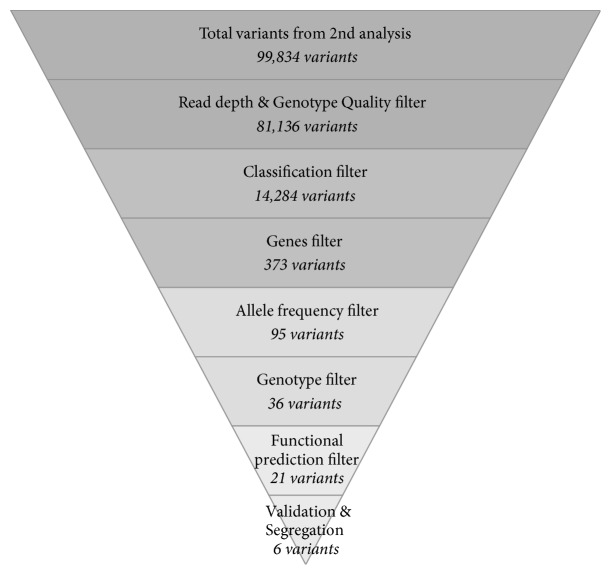
Filtering procedure of variants obtained by whole exome sequencing 2nd data analysis.

**Figure 4:**
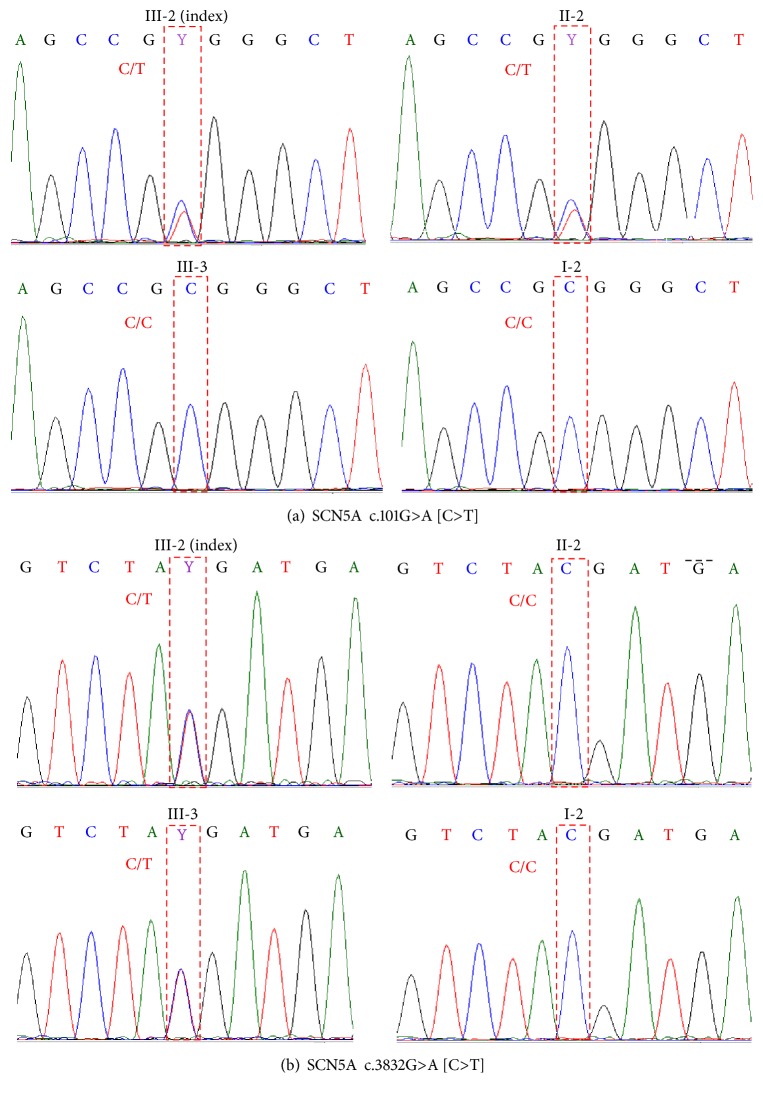
Chromatograms of 2 heterozygous missense variants in* SCN5A* gene: c.101G>A (a) and c.3832G>A (b). Letters in [] indicate complementary (FWD) alleles.

Two nonsynonymous missense variants in the* SCN5A* gene in this study, c.101G>A and c.3832G>A (NM_000335), were likely to be present in a compound heterozygous fashion because 2 heterozygous variants were found in same gene in the affected case but only one or none of them were found in unaffected family members. Heterozygous c.101G>A was found in III-2 (index) and II-2 (mother) while heterozygous c.3832G>A was found in III-2 (index) and III-3 (brother) (Figure 2). Chromatograms of both variants in all available subjects are shown in Figure 4.

All other 4 variants were nonsynonymous missense variants in which their genotypes were heterozygous. These variants consisted of c.28730C>T (NM_133378) in the* TTN* gene, c.449C>T (NM_153240) in the* NPHP3* gene, c.1133A>T (NM_012144) in the* DNAI1* gene, and c.13481A>C (NM_002458) in the* MUC5B* gene.* TTN* encoded Titin or connectin, a giant muscle protein, is expressed in the cardiac and skeletal muscles.* NPHP3* encodes a protein that is required for normal ciliary development.* DNAI1* encodes a member of the dynein intermediate chain family. Lastly,* MUC5B* encodes a protein member of the mucin family.

Finally, incidental findings in the whole exome data in this family following ACMG recommendations were explored. Aside from variants in the* SCN5A* gene that were assigned in the ACMG panel as known/expected pathogenic variants for Romano-Ward Long QT syndromes Types 1, 2, and 3 and Brugada syndrome, which were key variants in this family, a variant in the* MYBPC3* gene that was assigned in the ACMG panel for hypertrophic/dilated cardiomyopathy was explored and validated.

## 4. Discussion

Although a congenital heart defect was found in this autistic patient, this was unlike the Timothy syndrome which is a rare disorder that affects heart, nervous system, and fingers/toes. Syndactyly, the webbing of fingers and toes, one of Timothy syndrome signs, was not found in this case. Moreover, by direct sequencing and whole exome sequencing, variants in the* CACNA1C* gene, which are the common causes of both classical and atypical Timothy syndrome, were not detected [25]. Therefore, it was inferred that heart block and autism in this family are a coincidence.

**Figure 5:**
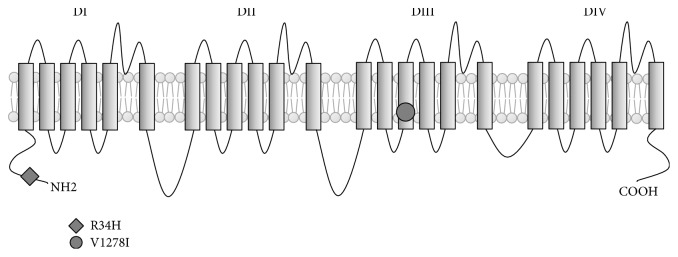
Schematic of the transmembrane topology of the* SCN5A* protein. The location of the variants R34H (c.101G>A) and V1278I (c.3832G>A) is shown.

*SCN5A*, a cardiac sodium channel gene, is important in generation and transmission of electrical impulses by its role in controlling the flow of sodium ions into cells [11]. Several variants in the* SCN5A* that are located in four homologous domains of alpha subunit of NaV1.5, including 6 segments of each domain and linkers, N-terminal, and C-terminal, have been reported to be associated with various phenotypes of cardiac diseases [26]. The mechanism of* SCN5A* mutations associated with cardiac conduction disease has been explained by loss of function in NaV1.5 channels. Loss of NaV1.5 function leads to a reduction of inward sodium flow in the cells that results in difficulty producing and transmitting electrical signals. Conduction defects, however, could occur apart from transmission of inward sodium. The mutations on* SCN5A*, p.R219H, p.R222Q, and p.R225W which express alternative pathways, through a cation leak through NaV1.5, have been reported to be associated with mixed arrhythmias and dilated cardiomyopathy [27, 28]. In this study, 2 missense variants in the* SCN5A* gene in a patient who had complete heart block (III-2) were identified. The first variant, c.101G>A, results in replacement of arginine by histidine (p.R34H) in exon 2 whereas the second variant, c.3832G>A, results in replacement of valine by isoleucine (p.V1278I) in exon 21. Both variants were rare variants in which alternate alleles were not found in the Asian population from 1000 genome databases and the Thai population from the Thai in-house exome database. Functional prediction results show that both variants were predicted to alter protein function which indicates that these variants have a high possibility to damage function of the sodium channel and cause the conduction defect in this case (Table 3).

c.101G>A (p.R34H) is a novel variant which is located in the N-terminal cytoplasmic domain of the sodium channel alpha subunit (Figure 5). A variant (c.80G>A) in the same region, the N-terminal cytoplasmic domain of sodium channel, has been reported to be associated with Brugada syndrome by Priori et al. [29]. Makita et al. have reported a mutation, which resulted in a stop codon (p.Q55X), presented in a Brugada syndrome affected patient with 1st-degree AV block history [30].

c.3832G>A (p.V1278I) has been reported as a “disease causing mutation” of dilated cardiomyopathy in the HGMD database [31–33]. This variant is located in the S3 transmembrane segment of domain III (DIII) of the sodium channel alpha subunit. McNair et al. have reported that heterozygous c.3823G>A variant, which is located near c.3832G>A, was associated with dilated conduction disorder, cardiomyopathy, and arrhythmia [34]. In the same region, an association between atrial standstill and p.D1275N with polymorphisms in other gap junction protein, connexin40, has been reported by Groenewegen et al. [35]. Electrophysiological studies in xenopus oocytes showed that the c.3823G>A mutation results in an activation curve shift of the sodium channel conductance [35]. This activation curve shift toward more positive voltages may result in reduced excitability of myocytes. According to studies of Gosselin-Badaroudine et al. and Moreau et al., this electrical disturbance could be the result from positive charge leakages of mutated NaV1.5 channels that could lead to a Na+ leak into cardiac myocytes [27, 28].

Results from segregation analysis indicated that both variants in the* SCN5A* gene were most possibly inherited in a compound heterozygous manner. Heterozygous c.101G>A was present in family members III-2 (index) and II-2 but was absent in family members I-2 and III-3. Likewise, heterozygous c.3832G>A was present in family members III-2 (index) and III-3 but was absent in family members I-2 and II-2 while only III-2 was the heart block affected family member. Although most of reported variants in the* SCN5A* gene were autosomal dominant, however, variants that were inherited in compound heterozygous individuals, similar to the current case finding, have been previously reported.

For instance, Bezzina et al. described compound heterozygous inheritance of 2 variants in the* SCN5A* gene which was associated with severe cardiac conduction disturbances and degenerative changes in the conduction system [36]. A nonsense p.W156X, which is located in the S1-S2 linker of domain I, was inherited from the father and a missense p.R225W, which was located in S4 segment of domain I, was inherited from the mother [36].

Benson et al. studied compound heterozygous variants in* SCN5A* in three families with congenital sick sinus syndrome [13]. A heterozygous missense p. P1298L, which is located in S4 segment of domain III, and p.G1408R, which is located in S5-S6 linker of domain III, were found in three siblings of the first family. Heterozygous missense p.T220I and p.R1623X were found in an individual of the second family. These two variants were located in the S4 segment of domain I and S4 segment of domain IV. The third family presented heterozygous deletions p.delF1617 in S3-S4 linker of domain IV and the missense p.R1632H in S4 segment of domain IV [13].

Beside the variants in* SCN5A* gene, 4 heterozygous missense variants in* TTN*,* NPHP3*,* DNAI1,* and* MUC5B* genes were found in the present case. All variants were found in only the index case, family member III-2. Among these 4 genes, TTN was the most likely cardiac disease associated gene while lack of evidence for* NPHP3*,* DNAI1,* and* MUC5B* genes existed. Mutations in* TTN* have been reported in about 18% of sporadic dilated cardiomyopathies and 25% of familial autosomal dominant cardiomyopathies and rarely caused hypertrophic cardiomyopathies [37].

Since this study only focused on the coding variants in the exon by whole sequencing, noncoding variants and structural variants were not explored. Moreover, it should be noted that novel variants in the study were found by the bioinformatics process so determination of biological functions will be needed in further studies.

## 5. Conclusion

In conclusion, this study demonstrated the potential of whole exome sequencing and a bioinformatics pipeline to identify the possible causative variants of complete heart block in a Thai family. The investigation found compound heterozygous variants in the* SCN5A*, cardiac sodium channel subunit gene, of which one was a novel variant and another one was a known pathogenic variant. Additionally, a heterozygous missense variant in the* TTN*, titin or connectin gene, has also been identified.

## Figures and Tables

**Table 1:** List of genes involved in cardiovascular system defects.

*AARS2*	*ATP6V0A2*	*CSRP3*	*FASTKD2*	*HAND1*	*LMF1*	*MYH11*	*PLN*	*SFTPA1*	*TGFB2*
*ABCA1*	*ATRX*	*CTF1*	*FBLN5*	*HAND2*	*LMNA*	*MYH6*	*PLOD1*	*SFTPA2*	*TGFB3*
*ABCA3*	*B3GAT3*	*CTNNA3*	*FBN1*	*HCN4*	*LPIN1*	*MYH7*	*PNPLA3*	*SFTPB*	*TGFBR1*
*ABCC6*	*BAG3*	*DES*	*FBN2*	*HERG*	*LPL*	*MYL2*	*PPARA*	*SFTPC*	*TGFBR2*
*ABCC9*	*BCOR*	*DHCR24*	*FGD1*	*HFE*	*LRP5*	*MYL3*	*PPARG*	*SFTPD*	*THEMIS*
*ABCG5*	*BMPR2*	*DHCR7*	*FGFR2*	*HOXA1*	*LTBP4*	*MYLK*	*PPP1R17*	*SGCD*	*TLL1*
*ABCG8*	*BRAF*	*DLL3*	*FHL1*	*HRAS*	*MAP2K1*	*MYLK2*	*PRDM16*	*SGCG*	*TMEM43*
*ACADVL*	*CACNA1B*	*DMD*	*FHL2*	*IGBP1*	*MAP2K2*	*MYO6*	*PRKAB2*	*SHOC2*	*TMEM70*
*ACTA1*	*CACNA1C*	*DMPK*	*FKTN*	*ILK*	*MED12*	*MYOCD*	*PRKAG2*	*SKI*	*TMPO*
*ACTA2*	*CACNA1D*	*DNAH11*	*FLNA*	*IRX4*	*MEF2C*	*MYOM1*	*PRKAR1A*	*SLC25A3*	*TNNC1*
*ACTC*	*CACNA2D1*	*DNAH5*	*FLNB*	*JAG1*	*MHY11*	*MYOT*	*PRKG1*	*SLC25A4*	*TNNI3*
*ACTC1*	*CACNB2*	*DNAI1*	*FOXC2*	*JPH2*	*MIB1*	*MYOZ2*	*PSEN1*	*SLC2A10*	*TNNT2*
*ACTN2*	*CALM1*	*DNAJC19*	*FOXH1*	*JUP*	*MID1*	*MYPN*	*PSEN2*	*SLMAP*	*TNXB*
*ACVR2B*	*CALM2*	*DNM1L*	*FOXRED1*	*KCNA5*	*MKKS*	*NEBL*	*PTPN11*	*SMAD3*	*TOPBP1*
*ACVRL1*	*CALR3*	*DOLK*	*FRYL*	*KCND3*	*MKS1*	*NEXN*	*PTRF*	*SMAD4*	*TPM1*
*ADCK3*	*CASQ2*	*DPP6*	*FXN*	*KCNE1*	*MOG1*	*NF1*	*RAF1*	*SMAD9*	*TPM2*
*ADRB1*	*CAV1*	*DSC2*	*GAA*	*KCNE1L*	*MRPL3*	*NIPBL*	*RAI1*	*SNTA1*	*TRDN*
*AGL*	*CAV3*	*DSG2*	*GATA4*	*KCNE2*	*MTND1*	*NKX2.5*	*RANGRF*	*SNX3*	*TRIM63*
*AKAP9*	*CBL*	*DSP*	*GATA5*	*KCNE3*	*MTND5*	*NKX2.6*	*RASA1*	*SOS1*	*TRPM4*
*AKT3*	*CBS*	*DTNA*	*GATA6*	*KCNH2*	*MTND6*	*NKX2-5*	*RBM10*	*SOX2*	*TSFM*
*ALMS1*	*CFC1*	*EFEMP2*	*GATAD1*	*KCNJ2*	*MTTD*	*NODAL*	*RBM20*	*SOX7*	*TTN*
*ALPK3*	*CHD7*	*EIF2AK4*	*GATT6*	*KCNJ5*	*MTTG*	*NOS1AP*	*RET*	*SPEG*	*TTR*
*ANGPTL3*	*CHST14*	*ELMOD2*	*GDF1*	*KCNJ8*	*MTTH*	*NOTCH1*	*RPL4*	*SPRED1*	*TWIST1*
*ANGPTL4*	*COA5*	*ELN*	*GDF2*	*KCNK3*	*MTTI*	*NOTCH2*	*RPSA*	*SURF1*	*TXNRD2*
*ANK2*	*COL18A1*	*EMD*	*GJA1*	*KCNQ1*	*MTTK*	*NOTCH3*	*RYR1*	*SYNE1*	*UQCRB*
*ANKRD1*	*COL1A1*	*ENG*	*GJA5*	*KRAS*	*MTTL1*	*NPC1*	*RYR2*	*SYNE2*	*USF1*
*ANO5*	*COL1A2*	*EPHX2*	*GLA*	*LAMA4*	*MTTL2*	*NPHP3*	*SALL1*	*TAZ*	*VCL*
*APOA1*	*COL2A1*	*ESCO2*	*GLB1*	*LAMP2*	*MTTM*	*NPPA*	*SALL4*	*TBX1*	*VCP*
*APOA2*	*COL3A1*	*EVC*	*GLI3*	*LBR*	*MTTP*	*NRAS*	*SCN1B*	*TBX20*	*VHL*
*APOA5*	*COL4A1*	*EVC2*	*GNAI2*	*LCAT*	*MTTQ*	*NSDHL*	*SCN2B*	*TBX3*	*XK*
*APOB*	*COL5A1*	*EYA1*	*GPC3*	*LDB3*	*MTTS1*	*NUBPL*	*SCN3B*	*TBX5*	*ZASP*
*APOC2*	*COL5A2*	*EYA4*	*GPD1L*	*LDLR*	*MTTS2*	*PCSK9*	*SCN4B*	*TCAP*	*ZFPM2*
*APOE*	*CREBBP*	*FANCA*	*GPIHBP1*	*LDLRAP1*	*MUC5B*	*PDLIM3*	*SCN5A*	*TCTN3*	*ZIC3*
*ARHGAP31*	*CRELD1*	*FANCC*	*GSN*	*LEFTY2*	*MYBPC3*	*PEX7*	*SCO2*	*TERC*	*ZMPSTE24*
*ARX*	*CRYAB*	*FANCD2*	*GUSB*	*LIPC*	*MYCN*	*PKP2*	*SDHA*	*TERT*	*ZNF469*
*ATP5E*	*CSF2RA*	*FANCE*	*HADH*	*LIPI*	*MYF6*	*PKP4*	*SEMA5A*	*TFAP2B*	

**Table 2:** Candidate variants from whole exome sequencing.

Position	Gene	Classification	Transcript	HGVS coding	dbSNP
Chr1: 13036736	*PRAMEF22*	Nonsyn SNV	NM_001100631	c.808T>A	rs202011965
Chr2: 179402104	*TTN*	Nonsyn SNV	NM_003319	c.72635G>A	—
Chr2: 179542464	*TTN*	Nonsyn SNV	NM_133378	c.30443C>T	—
Chr2: 179549988	*TTN*	Nonsyn SNV	NM_133378	c.28730C>T	rs146400809
Chr2: 203395591	*BMPR2*	Nonsyn SNV	NM_001204	c.1042G>A	rs201067849
Chr3: 38607905	*SCN5A*	Nonsyn SNV	NM_000335	c.3832G>A	rs199473341
Chr3: 38674698	*SCN5A*	Nonsyn SNV	NM_000335	c.101G>A	rs199473046
Chr3: 132438619	*NPHP3*	Nonsyn SNV	NM_153240	c.449C>T	rs142663818
Chr7: 42064927	*GLI3*	Nonsyn SNV	NM_000168	c.1292A>G	—
Chr9: 34506694	*DNAI1*	Nonsyn SNV	NM_012144	c.1133A>T	—
Chr9: 97080945	*FAM22F*	Del	NM_017561	c.2071_2073delTCT	rs150455117
Chr11: 1267969	*MUC5B*	Nonsyn SNV	NM_002458	c.9859A>C	—
Chr11: 1271591	*MUC5B*	Nonsyn SNV	NM_002458	c.13481A>C	rs201038498
Chr11: 47356616	*MYBPC3*	Unknown	NM_000256	c.2883C>T	—
Chr11: 126143258	*FOXRED1*	Frameshift Del	NM_017547	c.445delC	—
Chr12: 58177067	*TSFM*	Splicing	NM_001172695	c.231+1_231+2delGT	—
Chr12: 112892433	*PTPN11*	Stop-gain	NM_002834	c.591T>G	rs76982592
Chr16: 1245007	*CACNA1H*	Nonsyn SNV	NM_001005407	c.335T>C	—
Chr16: 71061495	*HYDIN*	Stop-loss	NM_017558	c.3052T>C	rs146649547
Chr16: 89815152	*FANCA*	Nonsyn SNV	NM_000135	c.3263C>T	rs17233497
Chr21: 35821734	*KCNE1*	Nonsyn SNV	NM_001127670	c.199C>T	rs199473645

**Table 3:** List of final candidate variants.

Genomic coordinates	Genotype^*∗*^				AA change	Gene	MAF		Functional prediction		
	III-2	III-3	II2	I2			1kG (ASN)	Thai	SIFT	Polyphen 2 HumVar	Mutation taster
Chr2: 179549988	A/G	G/G	G/G	G/G	p.Pro9577Leu	*TTN*	0.01	0	Damaging	Probably damaging	Disease causing
**Chr3: 38607905**	**C/T**	**C/T**	**C/C**	**C/C**	**p.Val1278Ile**	***SCN5A***	**0**	**0**	**Damaging**	**Probably damaging**	**Disease causing**
**Chr3: 38674698**	**C/T**	**C/C**	**C/T**	**C/C**	**p.Arg34His**	***SCN5A***	**0**	**0**	**Damaging**	**Possibly damaging**	**Disease causing**
Chr3: 132438619	A/G	G/G	G/G	G/G	p.Ala150Val	*NPHP3*	0.02	0.0067	Damaging	Probably damaging	Disease causing
Chr9: 34506694	A/T	A/A	A/A	A/A	p.Tyr378Phe	*DNAI1*	0	0	Damaging	Benign	Disease causing
Chr11: 1271591	A/G	A/A	A/A	A/A	p.Lys4494Thr	*MUC5B*	0	0.0067	Damaging	Benign	Polymorphism

^*∗*^Genotypes in this table are FWD genotype while genotypes in HGVS are REV genotype.
